# Variation in the flowering time orthologs *BrFLC* and *BrSOC1* in a natural population of *Brassica rapa*

**DOI:** 10.7717/peerj.1339

**Published:** 2015-11-30

**Authors:** Steven J. Franks, Beatriz Perez-Sweeney, Maya Strahl, Anna Nowogrodzki, Jennifer J. Weber, Rebecca Lalchan, Kevin P. Jordan, Amy Litt

**Affiliations:** 1 Department of Biological Sciences, Fordham University, Bronx, NY, United States of America; 2 Pfizer Laboratory, The New York Botanical Garden, Bronx, NY, United States of America; 3 Center for Education Outreach, Baylor College of Medicine, Houston, TX, United States of America; 4 Department of Genetics and Genomics, Mt. Sinai Hospital, New York, NY, United States of America; 5 Comparative Media Studies, Massachusetts Institute of Technology, Cambridge, MA, United States of America; 6 Department of Plant Biology, Southern Illinois University at Carbondale, Carbondale, IL, United States of America; 7 Department of Botany and Plant Sciences, The University of California, Riverside, CA, United States of America

**Keywords:** Candidate gene, Phenology, Gene expression, Climate change

## Abstract

Understanding the genetic basis of natural phenotypic variation is of great importance, particularly since selection can act on this variation to cause evolution. We examined expression and allelic variation in candidate flowering time loci in *Brassica rapa* plants derived from a natural population and showing a broad range in the timing of first flowering. The loci of interest were orthologs of the Arabidopsis genes *FLC* and *SOC1* (*BrFLC* and *BrSOC1*, respectively), which in Arabidopsis play a central role in the flowering time regulatory network, with *FLC* repressing and *SOC1* promoting flowering. In *B. rapa*, there are four copies of *FLC* and three of *SOC1*. Plants were grown in controlled conditions in the lab. Comparisons were made between plants that flowered the earliest and latest, with the difference in average flowering time between these groups ∼30 days. As expected, we found that total expression of *BrSOC1* paralogs was significantly greater in early than in late flowering plants. Paralog-specific primers showed that expression was greater in early flowering plants in the *BrSOC1* paralogs *Br004928, Br00393* and *Br009324*, although the difference was not significant in *Br009324*. Thus expression of at least 2 of the 3 *BrSOC1* orthologs is consistent with their predicted role in flowering time in this natural population. Sequences of the promoter regions of the *BrSOC1* orthologs were variable, but there was no association between allelic variation at these loci and flowering time variation. For the *BrFLC* orthologs, expression varied over time, but did not differ between the early and late flowering plants. The coding regions, promoter regions and introns of these genes were generally invariant. Thus the *BrFLC* orthologs do not appear to influence flowering time in this population. Overall, the results suggest that even for a trait like flowering time that is controlled by a very well described genetic regulatory network, understanding the underlying genetic basis of natural variation in such a quantitative trait is challenging.

## Introduction

Genetic variation contributes to phenotypic variation and provides the raw material that natural selection acts upon to produce adaptive evolution. Despite a burgeoning amount of genetic and genomic information, we still know little about genetic variation in ecologically important traits in natural populations. One such trait in plant populations is the timing of first flowering. Flowering time is a key life-history trait that influences mating opportunities, reproductive fitness, gene flow and evolution (Elzinga et al., 2007; Franks, 2015; Primack, 1985). With changing climatic conditions, there have been widespread shifts to earlier flowering (Miller-Rushing & Primack, 2008; Parmesan & Yohe, 2003), with important implications for population and evolutionary dynamics. Plant populations can potentially respond to climate change through migration, plasticity or evolution, although their ability to do so may be limited (Franks, Weber & Aitken, 2014). To predict the ability of populations to evolve in response to climate change, it is particularly useful to understand the relationship between genetic variation and phenotypic variation in the traits of interest, since selection can act on this variation to produce evolutionary change (Hoffmann & Sgro, 2011). Although the genetic basis of phenotypic variation and evolutionary responses to climate change is rarely known, this is an emerging area of investigation, with the genetic basis of variation in flowering time particularly amenable to study (Franks & Hoffmann, 2012).

To investigate the genetic basis of phenotypic variation and evolutionary changes in flowering time, it is useful to work with a system where the phenotype is highly variable, and such an evolutionary shift has been documented. A rapid evolutionary shift to earlier flowering was shown to occur following a multi-year late season drought in California in two populations of the annual plant *Brassica rapa* L. (Franks, Sim & Weis, 2007). Within 7 generations during the drought, average flowering time in the Arboretum population, which is the focus of this study, shifted an average of 8.5 days earlier (Franks, Sim & Weis, 2007). Furthermore, there was a broad range in flowering time for selection to act upon within populations. For example, in the Arboretum population, grown in a greenhouse, the earliest flowering individuals initiated flowering 34 days after germination, while the latest flowering individual began flowering 112 days after germination. Flowering time was shown to be heritable, so variation in this trait has some genetic basis (Franks, Sim & Weis, 2007). Subsequent work showed that early flowering plants have lower water use efficiency and flower at a smaller size and earlier developmental stage (Franks, 2011). However, the genetic basis of this rapid evolutionary change in flowering time, as well as the genetic basis of flowering time variation within populations, remained unknown.

To investigate the genetic basis of flowering time variation and evolution in natural populations of *B. rapa*, we took advantage of the fact that there is a substantial amount of information known about genes, pathways, and processes involved in determining flowering time, mainly from work with the closely related plant *Arabidopsis thaliana* (for reviews, see e.g., Amasino & Michaels, 2010; Bastow & Dean, 2003; Michaels, 2009; Simpson & Dean, 2002). In Arabidopsis, flowering time is controlled by a complex integrated genetic regulatory network (Boss et al., 2004; Mouradov, Cremer & Coupland, 2002; Putterill, Laurie & Macknight, 2004) that promotes flowering at an appropriate time under suitable conditions, and suppresses flowering under environmental conditions that indicate inappropriate times to flower, such as too early or too late in the growing season. This effective regulation is the result of the integration of inputs from several internal and external signals through key genes that activate or suppress the flowering-promotion regulatory network (Boss et al., 2004). Thus, environmental factors and genes interact to influence flowering time, with environmental conditions serving as cues that signal appropriate times to flower, and also conditions such as stresses potentially inducing flowering (Riboni et al., 2014; Wada & Takeno, 2010; Ying, Chen & Cai, 2014). Differences in the activity of these key integrator genes could potentially underlie flowering time variation in natural populations. Two of these key central regulatory flowering time genes in Arabidopsis are *FLC* (*FLOWERING LOCUS C*; (Yan et al., 2010)) and *SOC1* (*SUPPRESSOR OF OVEREXPRESSION OF CONSTANTS 1*; (Immink et al., 2012; Lee et al., 2004)), which are the focus of investigation in this study.

*FLC* is a MADS-box transcription factor that has been the subject of much research on flowering time regulation (Bastow et al., 2004; Lempe et al., 2005; Michaels & Amasino, 1999; Michaels & Amasino, 2001; Searle et al., 2006; Sheldon et al., 2000). In Arabidopsis, *FLC* suppresses flowering by repressing the expression of *SOC1* and *FT*, which both promote flowering (Hepworth et al., 2002; Michaels & Amasino, 1999). When *FLC* is downregulated through the appropriate combination of signals, the key inducers of flowering are upregulated, and flowering is initiated. *FLC* is one of only a few flowering time genes that has been shown to vary in natural populations (Caicedo et al., 2004; Korves et al., 2007; Lempe et al., 2005; Scarcelli & Kover, 2009; Slotte et al., 2009; Stinchcombe et al., 2004). For example, previous studies in Arabidopsis have found latitudinal clines in frequencies of alleles of *FLC* and in flowering time (Caicedo et al., 2004; Gazzani et al., 2003; McKay, Richards & Mitchell-Olds , 2003), as well as a strong association between variation in *FLC* and variation in flowering time in a diverse panel of natural accessions (Lempe et al., 2005). Another study with Arabidopsis accessions found that expression in *FLC* was correlated with flowering time, although no genetic variation at *FLC* was detected in that study (Schläppi, 2001). These findings suggest that variation in *FLC* alleles may potentially influence flowering time in natural populations.

*SOC1* is also a MADS-box gene that plays a central role in flowering time regulation (Immink et al., 2012). *SOC1* promotes flowering (Liu et al., 2008; Moon et al., 2003) by activating the floral meristem identity genes (Immink et al., 2012). Recent research has characterized the mechanisms by which *SOC1* interacts with other elements in the flowering time regulatory network in more detail, and has demonstrated that *SOC1* is a key hub in the flowering time regulatory network (Immink et al., 2012).

Most of this previous work investigating the flowering time genetic regulatory network has focused on *Arabidopsis*, which is in the same family (Brassicaceae) as *Brassica*. Researchers working with *Brassica* have confirmed that many of the same genes and networks operate in both taxa (Kole et al., 2001; Lagercrantz et al., 1996; Lin et al., 2005; Osborn et al., 1997; Schranz et al., 2002; Schranz et al., 2007; Tadege et al., 2001). The genome of *B. rapa* has been sequenced and extensively annotated (Wang et al., 2011), facilitating work on flowering time genes in this species.

In contrast to Arabidopsis, which contains only one copy each of *FLC* and *SOC1*, the *B. rapa* genome possesses four copies of *FLC* and three of *SOC1* (http://brassicadb.org/brad/). The four *BrFLC* genes (*BrFLC1, BrFLC2, BrFLC3, BrFLC5*) co-localize with flowering time QTL and have been shown to influence flowering time in an additive fashion in *B. rapa* (Kole et al., 2001; Li et al., 2009; Lou et al., 2007; Nishioka et al., 2005; Okazaki et al., 2007; Osborn et al., 1997; Schranz et al., 2002; Xiao et al., 2013; Zhao et al., 2010). In addition, studies have shown that allelic sequence variation, including splice site polymorphism, is correlated with transcript levels of *BrFLC* genes and with flowering time (Li et al., 2009; Yuan et al., 2009; Zhao et al., 2010). Overexpression of a *B. rapa SOC1* ortholog (referred to as *BrAGL20*) in *B. napus* caused early flowering, suggesting that the function of this gene may be conserved (Hong et al., 2013). Quantitative gene expression analyses also indicate that at least two of the *SOC1* orthologs may potentially play a role in flowering induction in *B. rapa* (Xiao et al., 2013).

In this study, we investigated the genetic basis of flowering time variation in plants derived from a natural population of *Brassica rapa*. Selection may have acted upon this underlying genetic variation to produce the evolutionary shifts to earlier flowering time observed previously (Franks, Sim & Weis, 2007). We focused on sequence and expression variation in orthologs of the key *Arabidopsis* flowering time regulatory genes *FLC* and *SOC1*, testing the hypothesis that such variation underlies the natural variation observed in flowering time. We investigated sequence variation in coding regions, introns, and upstream promoter regions in all paralogs of these genes, and quantified the expression of each paralog. We predicted that we would find lower *BrFLC* expression and greater *BrSOC1* expression in early compared to late flowering plants. We looked for associations between allelic and expression variation at these genes and variation in flowering time, focusing on a set of the earliest and latest flowering individuals from the natural population grown under common conditions.

## Materials & Methods

### Sample collection and growing conditions

Seeds of *Brassica rapa* were collected in bulk from the Arboretum population in Irvine, California in the spring of 2008. The permit is #19699-21901 from the UC Reserve System (RAMAS) for collecting seeds of Brassica rapa at the San Joaquin Marsh Reserve, the University of California, Irvine. The Arboretum population is located on the grounds of the University of California Arboretum, adjacent to a wetland, and was previously shown to have a broad range in flowering time and to have evolved earlier flowering time in response to a natural drought (Franks, Sim & Weis, 2007). To determine the optimum tissue and developmental stage to sample for comparative gene expression analyses, we grew one set of plants (set 1) in controlled conditions to characterize changes in gene expression over time and among leaves. Once we had identified the appropriate stage and leaf for sampling, we grew two additional sets of plants (sets 2 and 3) in controlled conditions for the early–late flowering comparisons. Set one consisted of 16 plants; sets 2 and 3 consisted of 225 seeds selected haphazardly from the collection, at least 200 of which survived to first flowering. Because these seeds were haphazardly selected from the collection, they varied in flowering time. The seeds were planted in Sunshine mix #1 (Sungro Horticulture, Vancouver, Canada) in pots 6 cm × 6 cm × 9 cm deep, watered daily and fertilized once per week with 14-14-14 fertilizer. The plants were grown on light carts and given light 24 h per day, which allows flowering because *Brassica rapa* is a long-day plant (Salisbury, 1963). We recorded date of emergence (defined as the opening of the seed coat and emergence of the radicle) and date of first flowering (defined as the opening of the bud and visibility of both stigma and anthers) for all plants. We selected the earliest and latest flowering plants for all analyses of the association between flowering phenotype and genotype or gene expression level.

### DNA and RNA extraction

We used set 1 plants for analysis of gene expression over time and among leaves, set 2 plants for comparative analysis of gene expression and sequence analysis of the coding regions of the genes, and set 3 plants for analysis of allelic variation in regulatory regions of our candidate genes. For set 1, we used a sterilized hole punch to collect leaf tissue from the first and second true leaf as soon as each leaf reached 2 cm in length, and every 4 days thereafter. Leaf discs were flash frozen in liquid nitrogen and stored at −80 ° C. The samples were ground in liquid nitrogen and RNA was extracted using the RNeasy Plant Mini kit (Qiagen, Venlo, Limburg) according to the manufacturer’s protocol. RNA was treated with DNAse (NEB, Ipswich, MA) to remove contaminant genomic DNA, and cDNA was synthesized from 1 µg of RNA using the Superscript II enzyme kit (Life Technologies, Norwalk, Connecticut, USA) with random hexamer primers.

For set 2, we collected ∼1 g of leaf tissue from the second true leaf of all plants 16 days from planting, before the plants had come into flower. Results from set 1 indicated that removal of this amount of leaf material did not alter flowering time (there was no difference in average flowering time in plants with tissue removed compared to control plants without tissue removed), and also that gene expression level at day 16 was a good predictor of expression at other times. The leaf tissue was immediately frozen in liquid nitrogen upon collection and then stored at −80 °C. After all plants had flowered, frozen samples from the 10 earliest and 10 latest flowering plants were selected. RNA was extracted and cDNA synthesized as above.

For set 3, which was used to evaluate regulatory sequence variability, leaf tissue was collected from all plants 16 days after planting and stored in silica gel at room temperature. After the plants had flowered, samples from the 20 earliest and 20 latest flowering plants were selected, and subsets (generally 10 each) of these were used for analyses. Samples were ground using a FastPrep (MP Biomedicals, Santa Anna, California, USA) and DNA was extracted using the DNeasy Plant Mini kit (Qiagen, Venlo, Limburg) according to the manufacturer’s instructions.

### DNA amplification

CLC Main Workbench, v.6.8.2 (http://www.clcbio.com/products/clc-main-workbench) and Primer 3 (http://bioinfo.ut.ee/primer3-0.4.0/primer3/) were used to design all primers described below (Table 1, Figs. S1 and S2).

**Table 1:** Primer information. Primer coordinates and reference sequences are from th e BRAD database (http://brassicadb.org/brad/) except *BrFLC5*, which is from Genbank. Primers in exons were used for quantitative and semi-quantitative pcr, while primers in other regions were used for DNA sequencing. Locations of the primer attachment sites relative to the reference sequence are given in Figs. S1 and S2.

Locus	Region	Name	Location	Sequence
*BrSOC1*	Promoter	2.p4928f	−1008,−990	ATGAAGGGAAAAAGATGTG
Bra004928		2.p4928r	−308,−291	CCGAAACAAAACAAACCA
*BrSOC1*	Promoter	7.p9324f	−1100,−1082	GGACATTTTCGACCATACT
Bra039324		7.p9324r	−275,−258	ACCCAAAAACCAAACCAA
*BrSOC1*	Promoter	16.pB0393f	−912,−894	TTTGCTCTTCCTTTTTGCT
Bra000393		16.pB0393r	−184,−167	TTCCTGGGGTTTGATTTT
*BrSOC1*	Promoter	15.pA0393f	−580,−562	CTCCTATATCTCTCTATCT
Bra000393		15.pA0393r	217,234	TTTCTCTCTTTCTCTCTC
*BrSOC1*	Exon6	67.c4928f	467,487	AGGAGAAAGCTCTAGCTGCAG
Bra004928	UTR	67.c4928r	817,842	ATTAGATTCTACAGAGGCAAGTATAC
*BrSOC1*	Exon6	69.c9324f	468,487	GGAGAAAGCTCTAGCTGCAG
Bra039324	UTR	69.c9324r	803.825	AACATCTAGGTAGGCAACTGTAG
*BrSOC1*	Exon6	71.c0393f	495,514	GAAACTCGCTGAAAAGTGGG
Bra000393	UTR	71.c0393r	826,847	AAGTGTATGAGAAATTGAGAAC
*BrFLC2*	Promotor	FLC2f2	−2083,−2064	ACAGGTGGTATGAGTAATGA
Bra028599		FLC2r2	232,250	AAAGAGAAGAGGAACGGAA
*BrFLC3*	Promotor	FLC3f	−1179,−1158	TTACTTACTGAGTTCAATTGGG
Bra006051		GL1725r	−68,−49	CGGTTCAAGTGGCCGGAGAT
*BrFLC5*	Promotor	FLC5f1	−2762,−2742	ACTGGCATCCGAACACCCATG
KBrH038M21		FLC5r2	−77,−57	GTCGCCGGAGAGACTAAGCGT
*BrFLC1*	Exon1	GL1132f	33,52	TGAGAACAAAAGTAGCCGAC
Bra009055	Exon4	GL1155r	3291,3310	GAACCCACACTTACATTATC
*BrFLC2*	Exon1	GL1132f	33,52	TGAGAACAAAAGTAGCCGAC
Bra028599	Exon4	GL1157r	1791,1810	GTCGACGCTTACATCAGAAT
*BrFLC3*	Exon1	GL1132f	33,52	TGAGAACAAAAGTAGCCGAC
Bra006051	Exon4	GL1156r	1991,2009	TGTCCACGCTTACACCACC
*BrFLC5*	Exon1	GL1132f	33,52	TGAGAACAAAAGTAGCCGAC
KBrH038M21	Exon4	GL1158r	3304,3323	ATCCACGCTTACATCATCAA
*BrFLC1*	Exon4	GL1036f	3278,3297	GGAATCAAATGTCGATAATG
Bra009055	Exon7	GL1125r	4290,4310	TTAAGCAGCGGGAGAGTYAC
*BrFLC2*	Exon4	GL1037f	1780,1799	TGTGGAATCAAATTCTGATG
Bra028599	Exon7	GL1125r	3238,3256	TTAAGCAGCGGGAGAGTYAC
*BrFLC3*	Exon4	GL1038f	1978,1999	GGAATCAAATGTCGGTGGTGTA
Bra006051	Exon7	GL1125r	2922,2941	TTAAGCAGCGGGAGAGTYAC
*BrFLC5*	Exon4	GL1039f	3293,3312	TGTGGAATCAATTGATGATG
KBrH038M21	Exon7	GL1125r	4859,4878	TTAAGCAGCGGGAGAGTYAC
*BrFLC1*	Intron 1	GL1319f	203,224	CTGGGGTTTTCCATTATTATTGT
Bra009055		GL1319r	2603,2626	GTATGTTAGGATCAAAACTACCAG
*BrFLC2*	Intron 1	GL1320f	211,230	TCCTTTATTTGCCCTTTTCG
Bra028599		GL1321r	1260,1288	CAAAATAAGTTAAGATCAAAACAACTAGC
*BrFLC3*	Intron 1	GL1322f	214,236	TTTATTAGCCTTTTAAGCTTCTG
Bra006051		GL1323r	1281,1308	ACAATTAATGTTAAGAACAAAACTACTA
*BrFLC5*	Intron 1	GL1325f	216,236	TGCCCTTTAAGCTTTCTTCTC
KBrH038M21		GL1326r	2584,2607	GAGATCAAAAGTCAAAACTACTTG
*BrFLC1*	Exon4	GL1099f	3273,3298	CTTGAGGAATCAAATGTCGATAATGT
*Bra009055*		GL1100r	3320,3341	GTTCTCAAGGTGTTCCTCCAGC
*BrFLC2*	Exon3,Exon4	GL1136f	1685,1796	AAGTAAGCTTGTGGAATCAAATTCTG
Bra028599	Exon4,Exon5	GL1137r	1858,1953	TCAACATTAGTTCTGTCTTCCTAGCTCTA
*BrFLC3*	Exon4	GL1101f	1978,1999	GGAATCAAATGTCGGTGGTGTA
Bra006051		GL1102r	2031,2052	AGAGAGAGGGCATTTTCAAGGA
*BrFLC5*	Exon4	GL1138f	3287,3309	CAAGCTTGTGGAATCAATTGATG
KBrH038M21		GL1139r	3338,3360	GGGCAGTCTCAAGGTGATCTTCT

Promoter regions and coding sequences were amplified and sequenced for all *BrFLC* and *BrSOC1* paralogs. The first intron of the *BrFLC* loci was also sequenced, as evidence from Arabidopsis suggests it contains cis-regulatory elements (Sheldon et al., 2002). In many cases, not all of the 10 early and 10 late flowering individuals produced good quality sequence data. We attempted resequencing of individuals that initially did not produce good results, often several times. However, if no genetic variation was found in other individuals that did produce good results, and we were able to obtain good sequence from several early and late flowering individuals, we did not proceed beyond the earlier attempts at resequencing for individuals that did not produce good results. In addition, for some genomic regions, some paralogs proved difficult to amplify and sequence, therefore results are only presented for those loci for which clean sequence data was obtained for at least 5 early and 5 late flowering individuals, although in most cases our samples sizes were closer to 10 early and 10 late flowering individuals.

PCR reactions were performed as follows. For the promoter regions of the *BrFLC* paralogs, PCR reactions were performed on genomic DNA (gDNA). We used Taq 2x master mix (M0270; New England Biolabs, Ipswich, Massachusetts, USA) with a dNTP concentration of 200 µM, and final magnesium concentrations varying depending on the reaction. We used the following reaction conditions: an initial denaturation at 95 °C for 5 min, 35 cycles of denaturation at 95 °C, 30 s; annealing at variable temperatures, 30 s; elongation at 72 °C, variable times, and a final extension of 72 °C for 10 min.

Coding sequences and the first intron of all four *BrFLC* paralogs were amplified from cDNA and gDNA, respectively, using either (1) EconoTaq Plus Green 2X Master Mix (Lucigen, Middleton, Wisconsin, USA) in a reaction mix consisting of 7.5 µL EconoTaq, 4.8 µL water, 0.75 µL 10 mM primers, and 1.2 µL cNDA or gDNA, or (2) high activity Taq (Pluthero, 1993) in a reaction mix consisting of 9.4 µL water, 0.2 µL high activity Taq, 1.5 µL buffer, 0.6 µL MgCl2, 0.6 µL dNTPs (New England Biolabs, Ipswich, Massachusetts, USA), 0.75 µL 10 mM primers, and 1.2 µL cDNA or gDNA. PCR conditions were 94 °C for 5 min, 34–38 cycles of 94 °C for 30 s, annealing at appropriate temperature for 30 s, 64 °C or 72 °C for one minutes, and a final extension of 64 °C or 72 °C for 10 min. DNA was visualized on a 1% agarose gel stained with ethidium bromide. The coding sequences of all four *BrFLC* paralogs are similar, with a single variable region in the middle. This region was used to design paralog-specific reverse and forward primers. The reverse gene-specific primers were used with forward primers that annealed at the 5′ end of the coding sequence and were not paralog-specific; similarly, the forward paralog-specific primers were used with universal 3′ reverse primers to amplify the 3′ region of the genes. In this fashion all four paralogs were amplified in two sections with a gap in the middle where the primers annealed. All products of correct size were sequenced in both directions at the DNA Analysis Facility of Yale University (http://dna-analysis.research.yale.edu/). Sequences were analyzed, trimmed, and assembled in Sequencher (GeneCodes, Ann Arbor, Michigan, USA).

For amplifications of the *BrSOC1* promoters, we used the following reaction conditions: an initial denaturation at 95 °C for 2 min, 32 cycles of denaturation at 95 °C, 30 s; annealing at variable temperatures, 30 s; elongation at 72 °C, variable times, and a final extension of 72 degrees for 10 min. We used 2–3 µM forward and reverse primers each. We used variable magnesium concentrations and Taq 2x master mix (M0270, New England Biolabs, Ipswich, Massachusetts, USA) to amplify a region of the *BrSOC1* paralog coding sequences, and NEBNext High-Fidelity 2X PCR master mix (New England Biolabs, Ipswich, Massachusetts) to amplify a region of the *BrSOC1* paralog promoters. DNA was visualized in 1% agarose gels pre-stained with GelRed dye (RGB-4103T; Phenix, Candler, North Carolina, USA).

We amplified the coding sequences of the *BrSOC1* paralogs using the same reaction mixes and cycling parameters as for the *BrFLC* paralogs, with appropriate annealing temperatures.

## DNA Sequencing and Alignment

Sanger sequencing was performed at Genewiz (http://www.genewiz.com), Cornell (http://www.biotech.cornell.edu) and Yale University (http://dna-analysis.research.yale.edu/). Promoters include sequence within 4 kb upstream of the gene transcription start site. Two regions of each of the *BrFLC* paralog promoters (within 4 kb upstream of the transcription start site), and one region of each *BrSOC1* paralog promoter regions (within 4 kb upstream of the transcription start site, and including key regulatory elements such as the predicted *BrFLC* MADS box binding site) were sequenced. The contigs were assembled in CLC Main Workbench 7.6.2 (http://www.clcbio.com) and manually edited using overlapping (forward and reverse) sequence reads. Alignment of each paralog was performed using MUSCLE (Edgar, 2004) in CLC Main using *BrFLC* reference sequences Bra009055 (*BrFLC2*), Bra028599 (*BrFLC3*), Bra006051 (*BrFLC5*) and *BrSOC1* reference sequences Bra000393, Bra039345, Bra004928 from the BRAD database (http://brassicadb.org/brad/). Exon 7 and the 3′ UTR were sequenced together to insure that a single paralog was amplified for qRT-PCR analysis and to confirm genome annotations. Alignments were visually inspected for proper codon alignment.

### Quantitative expression analyses

Quantitative real-time PCR (qRT-PCR) was performed to quantify expression using set 1 hole punch material (to identify appropriate tissue and developmental stage for further analyses) and from the material collected from the 10 earliest and 10 latest flowering individuals of set 2 (to quantify expression of *BrFLC* and *BrSOC1* paralogs in early- and late-flowering plants) on an ABI 7300 Real-Time PCR System (Life Technologies, Carlsbad, California, USA) using SYBR Green Master Mix (Life Technologies, Carlsbad, California, USA). Primers (Table 1) were designed using the ABI Primer Express program. The *Brassica rapa* serine/threonine-protein phosphatases *PP2a* catalytic subunit, which we determined to be expressed at a constant and appropriate level in our tissue samples (data not shown), was used as an endogenous control. Expression was quantified for all four *BrFLC* paralogs, the three *BrSOC1* paralogs, and the control using three technical replicates for each sample and gene. All primers had comparable efficiencies. Reaction mixes consisted of 12.5 µL FastStart Universal SYBR Green Master Mix (Roche Diagnostics, Indianapolis, Indiana, USA), 2.4 µL forward and reverse primers (2.5 nmole), 15 ng cDNA template, and 3.5 µL sterile water. Reactions were run using the standard relative quantification cycling parameters: 95 °C for 20 s followed by 40 cycles of 95 °C for 3 s and 60 °C for 30 s. Relative expression was calculated using the ΔΔ*C_T_* method using the 7300 System SDS Software provided with the 7300 Real-Time PCR System.

Approximately 3–5 hole punches were collected from leaf one and leaf two from the 16 plants of set 1. Expression of *BrFLC3*, which our preliminary analyses had shown to be strongly expressed, was quantified across all hole punches and leaves to determine a tissue and stage to sample. These results indicated that the second true leaf, collected 16 days after sampling, was appropriate for analysis of gene expression. The reason for this was that expression at this time was at or near peak, and was correlated with expression levels at other times (Fig. 3). For example, expression at day 16 and day 18 was highly correlated (*r*^2^ = 0.86, *p* = 0.0064).

Because *BrSOC1* expression analysis was initially performed using general primers that amplified all *BrSOC1* paralogs, a second set of expression analysis was performed with *BrSOC1* paralog-specific primers (Table 1). We used semi-quantitative PCR to determine if there was a difference in expression between early and late flowering plants. For these assays, we performed PCR using cDNA for each of the *BrSOC1* paralogs on the same set of early and late flowering plants as the qPCR assays, with one sample per plant and 10 replicates of early and 10 of late flowering plants. PCR reactions were run at 95 °C for 20 s followed by 36–38 cycles of 95 °C for 3 s and 60 °C for 30 s. Products were run on 1% agarose gels that included a ladder that served as a product size indicator as well as an intensity standard. The same amount of cDNA was used in each reaction and the same amount of product was loaded into each lane. Band intensity relative to the ladder was quantified from the gel image using the program GeneTools version 4.03 (Syngene, Frederick, Maryland). Quantified relative band intensity was used as our semi-quantitative measure of gene expression in these assays.

## Statistical Analyses

Differences in gene expression between early and late flowering plants were analyzed with ANOVA. Differences in allele frequencies between early and late flowering plants were analyzed with Fisher exact tests and Wald two sample test of proportions.

## Results

### Flowering phenology

The *Brassica rapa* plants from the natural California population exhibited a broad range in flowering time when grown in the lab. We were able to sample plants that flowered in the early and late ends of the flowering time distribution and that were well above and below the mean flowering time. We examined flowering time in set 2 and set 3 plants.

**Figure 1:**
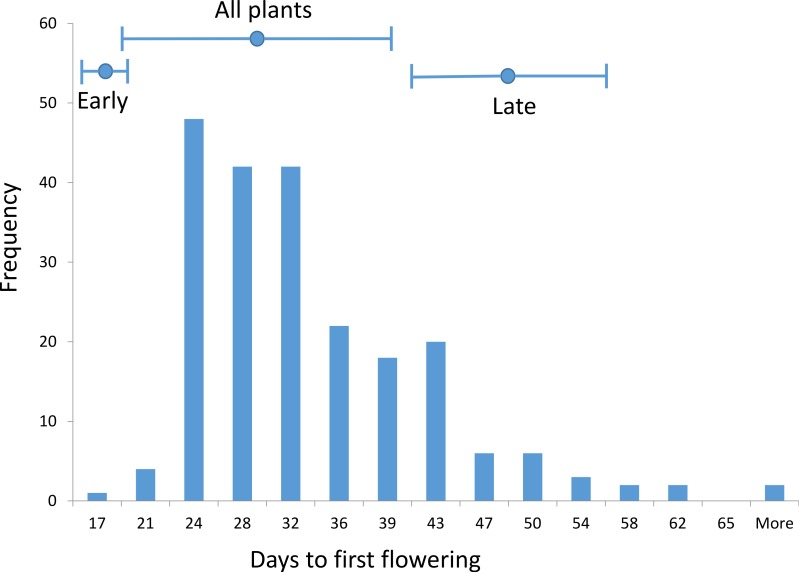
Flowering time. Shown is a histogram of flowering time for 203 *Brassica rapa* plants from the Arboretum population grown under common conditions on light carts in the lab. Above the histogram, the mean (dot) and standard deviation (bar) flowering time is shown for plants from the early flowering group, the late flowering group, and all plants. Plants from the early and late flowering groups were chosen for analyses.

For set 2 plants, the average time to first flowering was 31.4 (±9.3) days (standard deviation in parentheses) (Fig. 1). The average time to first flowering was 20.7 (±1.2) days in the 20 earliest flowering plants and 51.9 (±7.4) days in the 20 latest flowering plants (Fig. 1). For set 3 plants, the average time to first flowering was 35.0 (±8.5) days. The average time to first flowering was 24.8 (±0.6) days in the 20 earliest flowering plants and 53.3 (±6.4) days in the 20 latest flowering plants.

### Gene expression

**Figure 2:**
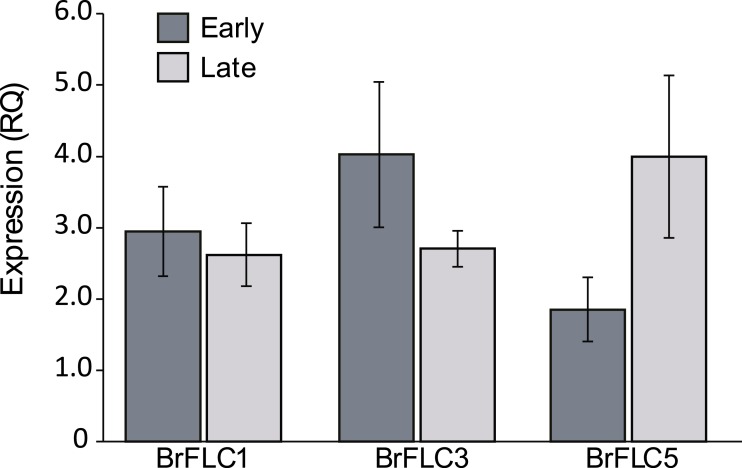
Expression of *BrFLC* genes. Shown are average relative expression (RQ) values from real-time quantitative PCR for the three *BrFLC* genes analyzed from individuals from the early (dark grey bars) and late (light grey bars) flowering groups. Bars represent 1 standard error.

**Figure 3:**
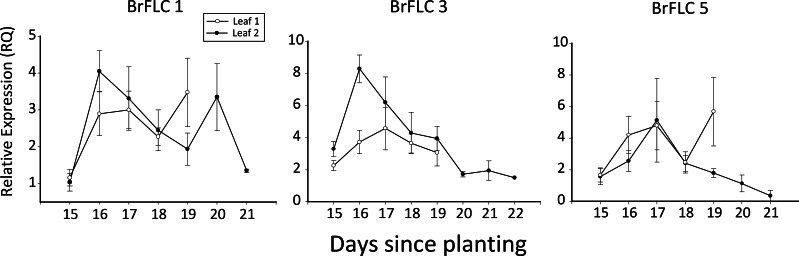
BrFLC expression over time. Shown are average relative expression (RQ) values from real-time quantitative PCR over time for the three *BrFLC* genes analyzed. Samples were taken from the first (white dots) and second (black dots) true leaves. The plants vary in flowering time. Bars represent 1 standard error.

For the *BrFLC* genes, we designed primers specific to each paralog for quantitative reverse transcription PCR (qRT-PCR) analysis. We did not obtain sufficient sequence data for *BrFLC2* for statistical evaluation. There was no difference in expression between early and late flowering plants (set 2) for *BrFLC1* (*F*_1,18_ = 0.18, *p* = 0.68), *BrFLC3* (*F*_1,17_ = 1.75, *p* = 0.20) or *BrFLC5* (*F*_1,7_ = 2.53, *p* = 0.16). There was also no difference in expression between early and late flowering plants for the expression of the three *BrFLC* genes summed together (*F*_1,18_ = 0.40, *p* = 0.54). Trends showed greater expression in early than late flowering plants for *BrFLC3*, and greater expression in late than early plants in *BrFLC5* (Fig. 2), but these were not statistically significant. There was variation in expression of the three *BrFLC* paralogs over time (set 1), with expression generally increasing at first and then showing an eventual decline, although there was variation in this pattern (Fig. 3).

**Figure 4:**
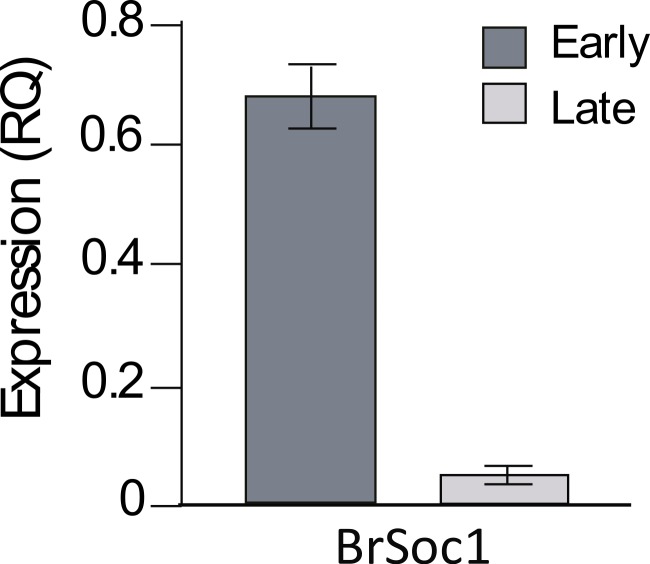
Total *BrSOC1* expression. Shown are average relative expression (RQ) values from real-time quantitative PCR for early (dark grey bars) and late (light grey bars) flowering plants using primers that amplified *BrSOC1* generally and were not paralog-specific. Bars represent 1 standard error.

**Figure 5:**
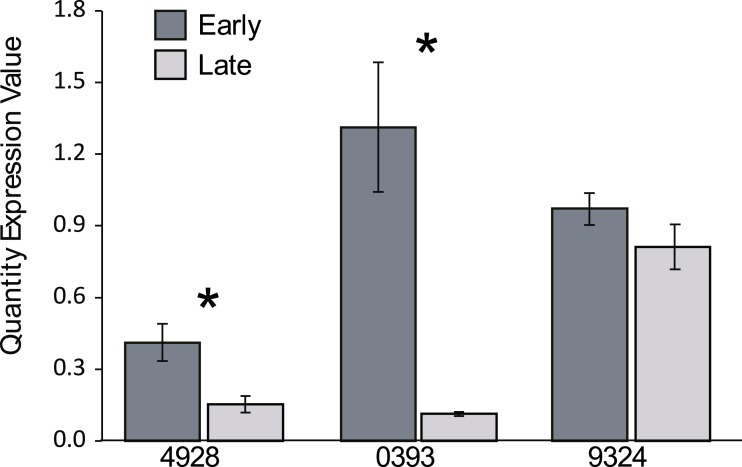
Paralog-specific *BrSOC1* expression. Shown are quantity expression values (QEV) derived from semi-quantitative pcr (see ‘Methods’ for details) for the three *BrSOC1* paralogs (Br004928, Br000393, Br009324) for plants from the early (dark grey bars) and late (light grey bars) flowering groups. Bars represent 1 standard error. An * indicates that expression of early and late flowering individuals was significantly different for a given paralog at *p* < 0.05.

For the *BrSOC1* genes, our initial primers amplified all paralogs together when used on cDNA prepared from set 2 plants. We found that combined *BrSOC1* expression was significantly greater in early compared to late flowering plants (*F*_1,18_ = 49.2, *p* < 0.0001; Fig. 4), consistent with experiments in Arabidopsis showing that *SOC1* promotes flowering (Immink et al., 2012). Paralog-specific primers were then designed and products quantified using semi-quantitative PCR. Expression was significantly greater in early compared to late flowering plants for Bra004928 (*t* = 3.03, *d*.*f*. = 18, *p* = 0.007) and Bra000393 (*t* = 4.44, *d*.*f*. = 18, *p* = 0.0003), but not for Bra039324 (*t* = 1.25, *d*.*f*. = 17, *p* = 0.230) (Fig. 5).

### Allelic variation

Based on cDNA and gDNA sequencing of set 2 and set 3 plants, promoter regions, first introns, and coding sequences of all of four *BrFLC* genes showed no allelic variation. Sequences across the promoter and entire coding sequence appear to be fixed for all four *BrFLC* paralogs in *B. rapa*. Thus allelic variation at these loci does not explain variation in flowering time. Promoter regions of the *BrSOC1* paralogs (set 3) did show allelic variation at several sites. In particular, Br009324 showed variation at 5 sites, with each of these sites a SNP with two alternate alleles. However, there was no statistically significant association between this allelic variation at any of the sites with flowering time variation (Table 2). No other regions sequenced showed variation, so these were not tested for associations with flowering time.

**Table 2:** Statistics table. Statistical tests of association between allelic variation for each polymorphic nucleotide site in the promoter of locus Br009324, one of the *BrSOC1* paralogs, and flowering time variation comparing the early to the late flowering plants. Numbers across the top refer to nucleotide sites relative to the start codon. Tests are Fisher exact tests and Wald two sample test for proportions. Shown are *p*-values for two-tailed tests.

	−952	−920	−904	−852	−639
Fisher	0.1189	0.4667	0.1189	0.1189	0.1189
Wald	0.0880	0.2908	0.0880	0.0880	0.0880

## Discussion

In this study, we were able to take advantage of variation in flowering time in plants from a natural population of *Brassica rapa* to explore the relationship between this phenological variation and allelic and expression variation at candidate flowering time loci. This genetic variation is important since selection can potentially act upon it to cause evolutionary changes in flowering time. We found a clear association between flowering time and expression in two *BrSOC1* paralogs, but not with any of the *BrFLC* paralogs, and no relationship between flowering time and allelic variation at any of these loci in their coding or promoter regions.

Expression of two of the three *BrSOC1* paralogs was greater in early than in late flowering plants. The trend for the third *BrSOC1* paralog was in the same direction, but was not statistically significant. It thus appears that, as in Arabidopsis (Immink et al., 2012) and other species (Fu et al., 2014; Lei et al., 2013; Preston, Jorgensen & Jha, 2014), early upregulation of the *BrSOC1* genes is indicative and predictive of early flowering in *B. rapa*. The cause of the differential expression in the *BrSOC1* genes between early and late flowering plants remains unknown, because we were not able to detect any association between flowering time and allelic variation within the *BrSOC1* promoter regions. We had hypothesized that variation in the promoter regions would influence flowering time, but this hypothesis was not supported. It is possible that some association could have been detected with a larger sample size, but a strong association would have been detected even with our modest sample. It is also possible that a region of the promoter that we did not sequence influences *BrSOC1* expression. The absence of a relationship between flowering time variation and promoter variation suggests that there is an alternative explanation for the observed difference in expression levels between early- and late-flowering plants. Expression may be influenced by the products of activating and repressing upstream transcription factors. Although *FLC* is known to suppress expression of *SOC1* in Arabidopsis (Hepworth et al., 2002), there was no relationship between expression of any of the *BrFLC* paralogs and flowering time in our study, suggesting that regulation of the *BrSOC1* paralogs by the *BrFLC* paralogs is not a likely factor in the patterns that we observed. Other possibilities include orthologs of *FT* or *FD*, which upregulate *SOC1* in Arabidopsis. However, our very preliminary investigations with *BrFT* paralogs did not uncover any genetic variation associated with flowering time variation, although we did find greater *BrFT* expression in early flowering than in late flowering plants. Additional possible explanations for the differences in expression are variation in potential enhancers that are not located within the 4 kb promoter region, or chromatin or DNA epigenetic modifications that would influence regulation but that are not detected with standard sequencing mechanisms.

Despite the fact that *FLC* is known to be a key regulator of flowering time in Arabidopsis, we found no association between flowering time and expression or allelic variation at any of the *BrFLC* orthologs in our population. It is worth noting that *FLC* operates though the autonomous and vernalization pathways, and the plants in our southern California population neither receive nor require vernalization to initiate flowering. If the vernalization pathway is not as important in populations that do not experience cold temperatures, then genes in this pathway might not play as large a role in influencing phenotypic variation in such populations. Such genes could influence variation in flowering time in temperate populations. They could also potentially be important in local adaptation, and may come under selection with changing environmental and climatic conditions.

Previous studies in *Brassica* species have detected flowering time QTLs, and some of these loci map to known flowering time genes (Axelsson, Shavorskaya & Lagercrantz, 2001; Lou et al., 2007). Other studies have shown associations between changes in expression of flowering time genes and flowering time phenotypes. For example, one recent study found that in *Ambrosia artemisiifolia*, expression of the orthologs of the genes *AP1*, *FT* and *SOC1* changed during the course of flowering, and the genes *CRY2* and *SPY* differed in expression between an early and a late flowering population (Li, Zhang & Liao, 2015). Other studies have shown that genetic variation in flowering time genes can influence the timing of flowering, but these genetic variants were generally major mutations that caused loss of function. For example, one study of *Arabidopsis thaliana* showed that variation between null and wild-type alleles of the gene *FRI*, along with interactions with *FLC*, resulted in a geographic cline in flowering time (Caicedo et al., 2004). Variation in these genes and their interactions was also found to influence flowering time in a broad survey of natural accessions of *A. thaliana* (Werner et al., 2005). We found associations between gene expression and flowering time, consistent with this previous work, but we did not find specific genetic variation that could be linked with flowering time variation.

## Conclusions

Genetic regulatory networks are often highly integrated and complex, and can potentially greatly diverge from a simple additive model of genetic effects. The flowering time genetic regulatory network in Arabidopsis is well studied and contains over a hundred genes, regulatory elements and transcription factors that all work in concert to control the timing of flowering. How variation in such complex networks as this influences phenotypic variation in natural populations is unknown. The fact that a particular gene is part of this regulatory network does not necessarily mean that allelic or expression variation at that gene is responsible for variation in flowering time in natural populations. For example, *BrFLC* is known to play a central role in the flowering time regulatory network, but variation at this gene did not seem to influence variation in flowering time in the population examined in our study. Understanding how genetic variation influences phenotypic variation in natural populations is an emerging area of investigation, and is key to predicting how traits will evolve. This will be useful, for example, in predicting how traits such as flowering time will respond to selection by changing climatic conditions.

## Supplemental Information

10.7717/peerj.1339/supp-1 Figure S1 *BrSOC1* primers Relative locations of primers designed for sequencing promotor orthologs (A–C) and Exon 6 (D) of *BrSoc1* flowering time genes in *Brassica rapa*. Solid blue arrow indicates the start codon. Where applicable, colors indicate matching sets of primers. For specific locations and primer sequence information, see Table 1.

10.7717/peerj.1339/supp-2 Figure S2 *BrFLC*primers Relative locations of primers designed for sequencing promotor regions (A) and coding regions (B) of *BrFLC* flowering time genes in *Brassica rapa*. Solid blue arrow indicates the start codon. Where applicable, colors indicate matching sets of primers. (Note for FLC2, the primer set shown in green covers Exon 3 (forward) and Exon 5 (reverse); for FLC1, FLC3 and FLC5 this set covers only Exon 4.) For specific locations, corresponding primer names and sequence information, see Table 1.

10.7717/peerj.1339/supp-3 Supplemental Information 1 Accession numbers for BrFLC paralogs

## Additional Information and Declarations

### Field Study Permissions

The following information was supplied relating to field study approvals (i.e., approving body and any reference numbers): The permit is #19699-21901 from the UC Reserve System (RAMAS) for collecting seeds of Brassica rapa at the San Joaquin Marsh Reserve, the University of California, Irvine.

### DNA Deposition

The following information was supplied regarding the deposition of DNA sequences: The sequences can be found in the Supplemental Information.
