# Supplementary figures and images for: Variation in the flowering time orthologs BrFLC and BrSOC1 in a natural population of Brassica rapa
Source: PeerJ. 2015 Nov 30;3:e1339. doi: 10.7717/peerj.1339 (PMC4671188; doi:10.7717/peerj.1339)

### S1a *Br000393*

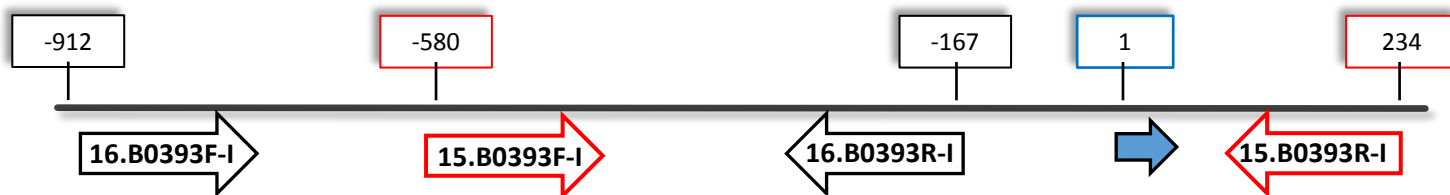

### S1b *Br004928*

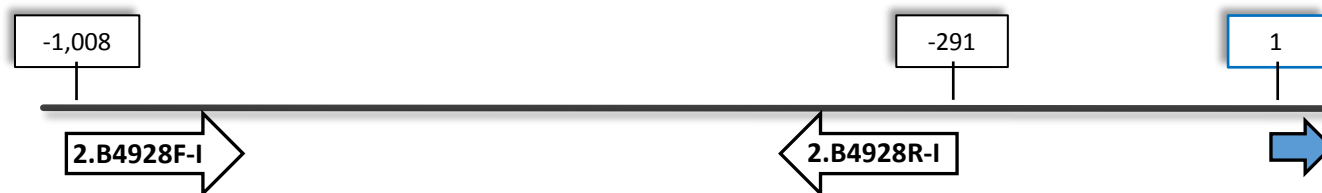

### S1c *Br0039324*

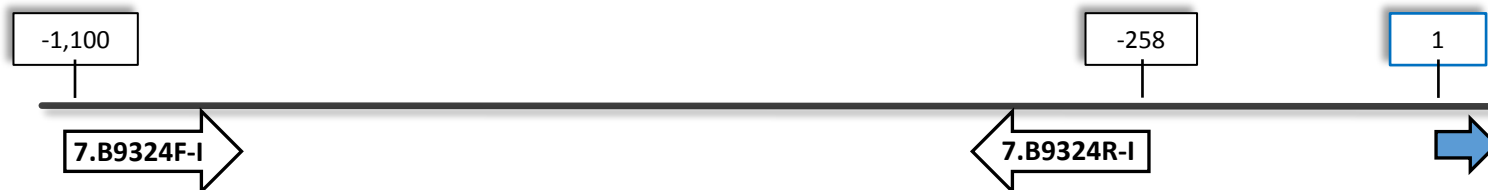

### S1d Exon 6, UTR

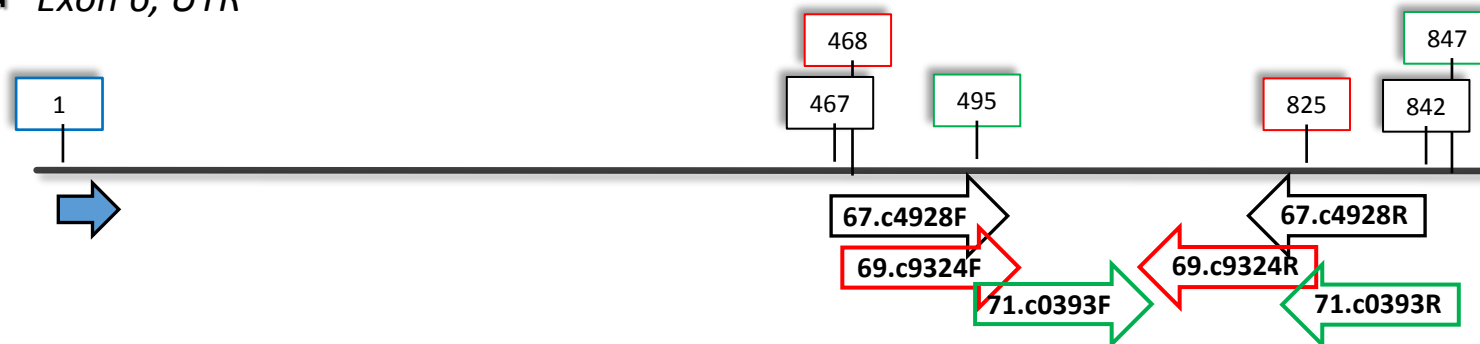

Supplement: Figure S1 — Relative locations of primers designed for sequencing promotor orthologs (A–C) and Exon 6 (D) of BrSoc1 flowering time genes in Brassica rapa. Solid blue arrow indicates the start codon. Where applicable, colors indicate matching sets of primers. For specific locations and primer sequence information, see Table 1. [file peerj-03-1339-s001.pdf]

## S2a *BrFLC* promoter primers

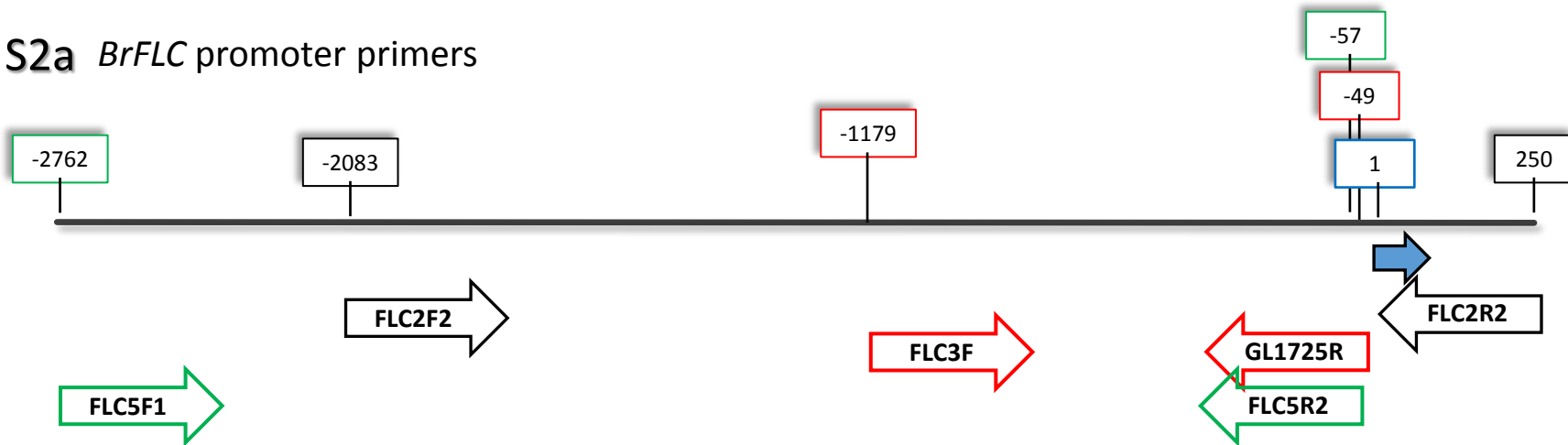

## S2b *BrFLC* coding primers

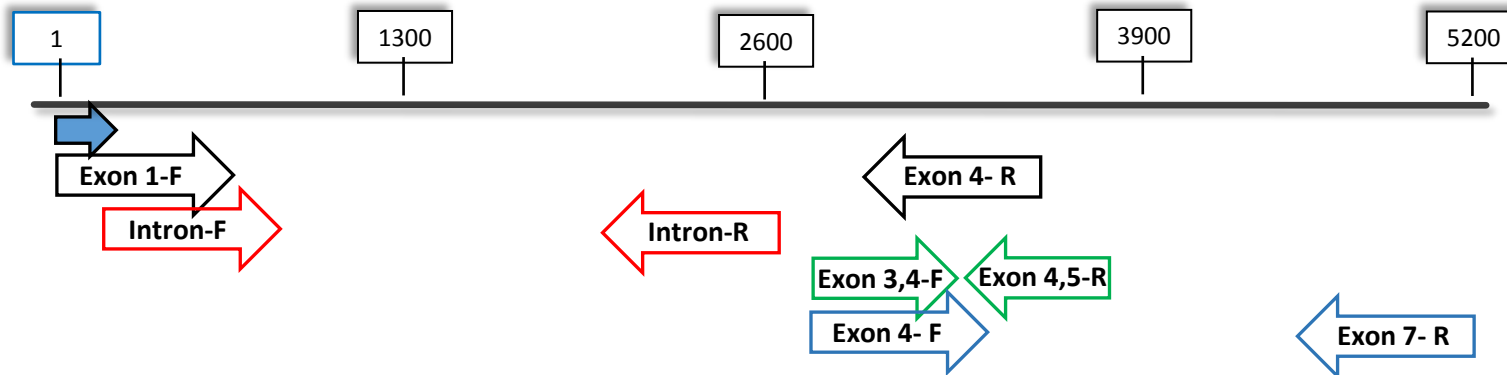

Supplement: Figure S2 — Relative locations of primers designed for sequencing promotor regions (A) and coding regions (B) of BrFLC flowering time genes in Brassica rapa. Solid blue arrow indicates the start codon. Where applicable, colors indicate matching sets of primers. (Note for FLC2, the primer set shown in green covers Exon 3 (forward) and Exon 5 (reverse); for FLC1, FLC3 and FLC5 this set covers only Exon 4.) For specific locations, corresponding primer names and sequence information, see Table 1. [file peerj-03-1339-s002.pdf]
